# 969. Gamification for Infectious Diseases Medical Education: Creating a Videogame to Teach COVID 19 Diagnosis and Treatment to Medical Students

**DOI:** 10.1093/ofid/ofab466.1164

**Published:** 2021-12-04

**Authors:** Gregory E Souza, Flávio Henrique Batista de Souza, Marconi A Aguiar dos Reis, Raoni A Dorim, Elisângela C Cristine Oliveira Gonçalves, Ana Luiza Silva Floriano, Bruna de Souza Tavares, André Magalhães de Oliveira, Daniela Da Guarda Ribeiro, Felipe Gonçalves de Sá, Júlia O Lacerda, Leandra Vieira Caetano, Ludmila Rodrigues Augusto, Maria Luiza Costa Santos, Otávio Reis Viana, Alef Santos Soares, Felipe Ferreira de Medeiros, Amanda Luiza Alves Figuereido, Sergio Alfredo Hidalgo Calderón, María José Rojas Puell, Andrea del Carmen Quispe Chauca, Maria Cristina Almeida, Arthur André Martins de Araújo, Artur Moreira Rodrigues, Cátia Ribeiro de Sá Rodrigues, Jefferson Ricardo Rodrigues Morais, Larissa Ludgero Soares Cardoso, Matheus Álvaro Colbert Câmara, Pedro Alcântara Antunes Lopes, Mateus Augusto Mendonça de Resende, João Pedro Amaral de Oliveira, Pedro Henrique Gonçalves de Souza, Izadora Serdeira de Souza, Samara Soares Leal, Mariana Aguiar, Maria Luiza Brandão de Faria, Paula G Ferreira, Leonora de Oliveira Rocha, Fernanda Luíza Pinheiro Montes, Luiz Felipe Oliveira Rocha

**Affiliations:** 1 Centro Universitário de Belo Horizonte - UniBH, Belo Horizonte, Minas Gerais, Brazil; 2 Centro Universitário de Belo Horizonte, Belo Horizonte, Minas Gerais, Brazil; 3 Belo Horizonte, Minas Gerais, Brazil; 4 Centro Universitário Atenas, Sete Lagoas, Minas Gerais, Brazil; 5 Universidade Federal de Viçosa, Belo Horizonte, Minas Gerais, Brazil; 6 Universidad Cientifica del Sur, Lima, Lima, Peru; 7 Centro Universitário UNIBH, Belo Horizonte, Minas Gerais, Brazil; 8 Centro Universitário de Belo Horizonte UniBH, Belo Horizonte, Minas Gerais, Brazil; 9 Centro Universitario De Belo Horizonte - UNIBH, Belo Horizonte, Minas Gerais, Brazil

## Abstract

**Background:**

Brazillian authorities reported a total of 16.3 million cases and 454.000 deaths during COVID-19 pandemic in Brazil by may 2021. It became necessary to educate healthcare professionals on diagnosis and treatment of the syndrome. Game based learning surfaced as an effective alternative, since it promotes critical thinking and problem solving skills. A team of Brazilian and Peruvian students, physicians, designers and programmers gathered to create a decision based computer game that simulates a hospital scenario and allows medical students to analise, make decisions and receive feedback. This work describes the creative process and showcase the initial version of the software.

**Methods:**

Professors and students of Medicine, Information Technology (IT), Design and Architecture from Brazil and Peru assembled a team in order to develop the computer game. Clinical cases were created by the medical students and professors, comprising medical procedures for the treatment and management of COVID 19, and a video game script was developed exploring gamification principles of challenge, objectivity, persistence, failure, reward and feedback. Algorithms (image 1) were created, under supervision of professors of Medicine, to define possible courses of action and outcomes (*e.g.* gain or loss of points, improvement or worsening of the patient). Students of Design created artistic elements, and IT students programmed with a game engine software.

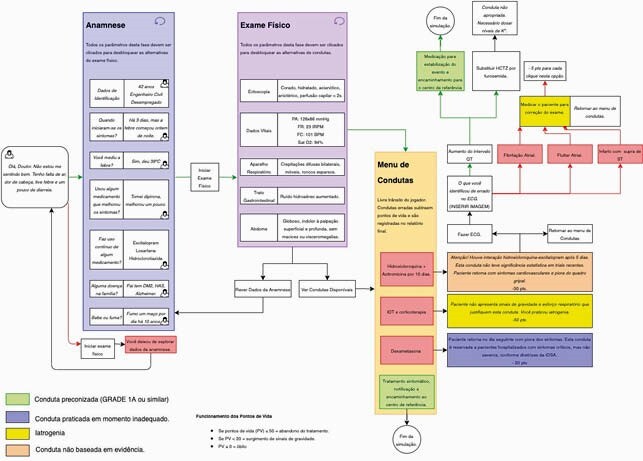

This fluxogram, written in portuguese, describes in detail all the possible courses of actions that can be exercised by the player. It is created by a team of Professors of Medicine and medical students, in accordance with evidence-based guidelines. Primarily, this document guides the programmers and designers throughout the development phase of the game.

**Results:**

Initially, an expandable minimum viable product was obtained. The game, visualized on image 2, consists in a non-playable character and a playable character (*i.e.* doctor), with a scenario and a dialogue script simulating a clinical examination of a COVID 19 patient. The player can interact with certain elements within the game, *e.g.* the computer and other characters, to retrieve test results or start dialogues with relevant information.

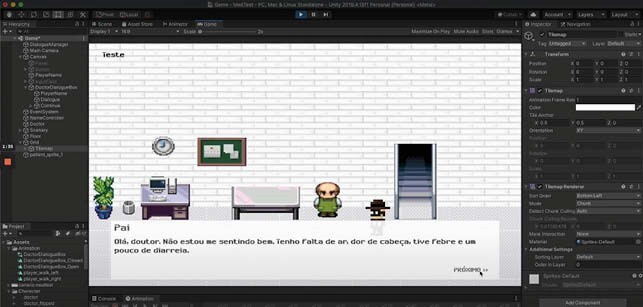

Hospital scenario and dialogue window between doctor (player in black) and patient (non playable character) are displayer in the game engine software (Unity 2D). On the bottom half of the screen, the dialogue box allows the player to collect the patient’s medical history. The player can interact with certain elements to obtain relevant information to make decision and progress in the game.

**Conclusion:**

The game allows medical students to practice diagnosis and treatment of COVID 19. Future versions will include assessment reports of player’s actions, and a new score system will be implemented. New diseases will be incorporated in the gameplay to match the variety of scenarios offered by real hospitals and patients. Artificial intelligence will be employed to optimize gameplay, feedback and learning.

**Disclosures:**

**All Authors**: No reported disclosures

